# TGF-β signaling and tumor microenvironment dynamics in bladder cancer progression post-BCG therapy: a longitudinal single-nucleus RNA-seq study

**DOI:** 10.1186/s12885-025-15079-8

**Published:** 2025-11-10

**Authors:** Seo-Young Lee, Yun-Hee Lee, Tae-Min Kim, U.-Syn Ha

**Affiliations:** 1 Department of Medical Informatics, College of Medicine, The Catholic University of Korea, Seoul, Republic of Korea; 2 Cancer Research Institute, College of Medicine, The Catholic University of Korea, Seoul, Republic of Korea; 3 Department of Biomedicine & Health Sciences, Graduate School, The Catholic University of Korea, Seoul, Republic of Korea; 4 Department of Urology, College of Medicine, The Catholic University of Korea, Seoul, Republic of Korea

**Keywords:** Bladder cancer, BCG, Single-cell sequencing, TGF- β signaling, Macrophage, Fibroblast

## Abstract

**Background:**

Although non-muscle-invasive bladder cancer (NMIBC) frequently recurs and progresses despite BCG therapy, cellular mechanisms behind this remain unclear. Understanding the dynamics within the tumor microenvironment (TME) and identifying pathways associated with BCG resistance are crucial for improving NMIBC treatment.

**Methods:**

To elucidate transcriptional dynamics and key cellular interactions in the TME, we performed longitudinal single-nucleus RNA sequencing of 9 NMIBC cases with three pairs of treatment-naïve and disease progression samples. The resulting 58,037 nuclei were subject to cell type and subtyping annotations along with cell–cell interaction analyses.

**Results:**

Our analysis revealed marked interpatient heterogeneity in malignant cells, accompanied by copy number alterations associated with disease progression. TGF-β signaling emerged as a key pathway, showing gradual enrichment from pre-treatment to post-progression samples. Within the tumor microenvironment, we identified distinct subtypes including *LAMP3* + dendritic cells and *iCAF* fibroblasts that followed unique pseudotime trajectories linked to progression. Cell–cell interaction analysis further highlighted critical ligand–receptor pairs such as *DSC2–DSG2* and *ENG–BMPR2*, which were associated with poor clinical outcomes. These interactions may represent hypothesis-generating candidates that warrant further validation.

**Conclusions:**

This study highlights key cellular and molecular mechanisms underlying bladder cancer progression Post-BCG treatment. Through single-nucleus RNA sequencing, we identified critical TME subtypes, TGF-β signaling involvement, and crucial ligand-receptor interactions such as DSC2-DSG2 and ENG-BMPR2, which may serve as preliminary biomarkers requiring confirmation in larger independent cohorts. These findings offer valuable insights into enhancement of treatment strategies and patient outcomes.

**Supplementary Information:**

The online version contains supplementary material available at 10.1186/s12885-025-15079-8.

## Introduction

Bladder cancer is a prevalent malignancy with a significant impact on global cancer mortality [1]. The clinical course and prognosis of bladder cancer are influenced by various clinicopathological factors. Muscle invasion is a pivotal criterion for classifying bladder cancer into non-muscle-invasive bladder cancer (NMIBC) and muscle-invasive bladder cancer (MIBC) [2]. This distinction is crucial for determining the appropriate clinical management strategy, including the decision for bladder preservation or removal. The treatment objective for NMIBC is to prevent its progression to MIBC, with Bacillus Calmette-Guérin (BCG) therapy being the gold standard treatment for high-risk NMIBC patients [3, 4].

However, a notable challenge in NMIBC management is that a significant proportion of patients do not respond to BCG therapy and subsequently progress to MIBC [5], known as BCG-refractory NMIBC [6]. This clinical phenomenon complicates decision-making, as delayed cystectomy in these patients often results in poorer prognoses compared to those undergoing early cystectomy. Understanding the cellular dynamics underlying bladder cancer progression, particularly within the tumor microenvironment (TME), and identifying the mechanisms of BCG resistance and potential biomarkers are crucial for improving treatment decisions for NMIBC patients.

Bladder cancer is a molecularly heterogeneous disease. The consensus molecular classification of MIBC has six distinct subtypes. NMIBC molecular subtyping exemplified by frameworks like UROMOL, has highlighted Class 2a as a high-risk category with poor progression-free survival due to late cell cycle dysregulation and epithelial-to-mesenchymal transition (EMT) [7]. While these insights have advanced our understanding of BCG-treated bladder cancer, clinical implementation remains limited, underscoring the need for prospective validation to integrate molecular subtypes into personalized treatment strategies.

Emerging evidence highlights the role of TGF-β in post-BCG recurrence of NMIBC. Elevated expression of TGF-β3 has been associated with poor prognosis in bladder cancer [8], while TGF-β1 has been implicated in tumor progression and adverse outcomes across multiple cancer types [9]. Mechanistically, TGF-β promotes stromal remodeling and epithelial-to-mesenchymal transition, which generate an immune-excluded tumor microenvironment by preventing CD8 + T-cell infiltration [10]. This mechanism might also reduce the efficacy of immune checkpoint inhibitors such as anti-PD-L1 therapies. Targeting TGF-β in combination with immune checkpoint inhibitors could represent a promising therapeutic strategy for patients with BCG-resistant bladder cancer [11].

To analyze cellular dynamics contributing to disease progression of bladder cancers, we have previously performed NMF-base deconvolution to resolve the disease/cellular heterogeneity in identifying expression features associated with disease progression after BCG treatment.

In this study, after repeated tissue collection while longitudinally observing the clinical course, we conducted single-nucleus RNA sequencing (snRNA-seq) on nine bladder cancer (BLCA) samples to characterize the TME. Treatment-naïve specimens and recurred specimens after BCG instillation were obtained from all nine patients during the clinical observation period, enabling a comparison of before and after, as well as between responsive and refractory disease states. Our analysis revealed major cellular subtypes of the TME in bladder cancer and identified subtypes and their abundance during disease progression.

## Materials and methods

### Patients and samples

**Fig. 1:**
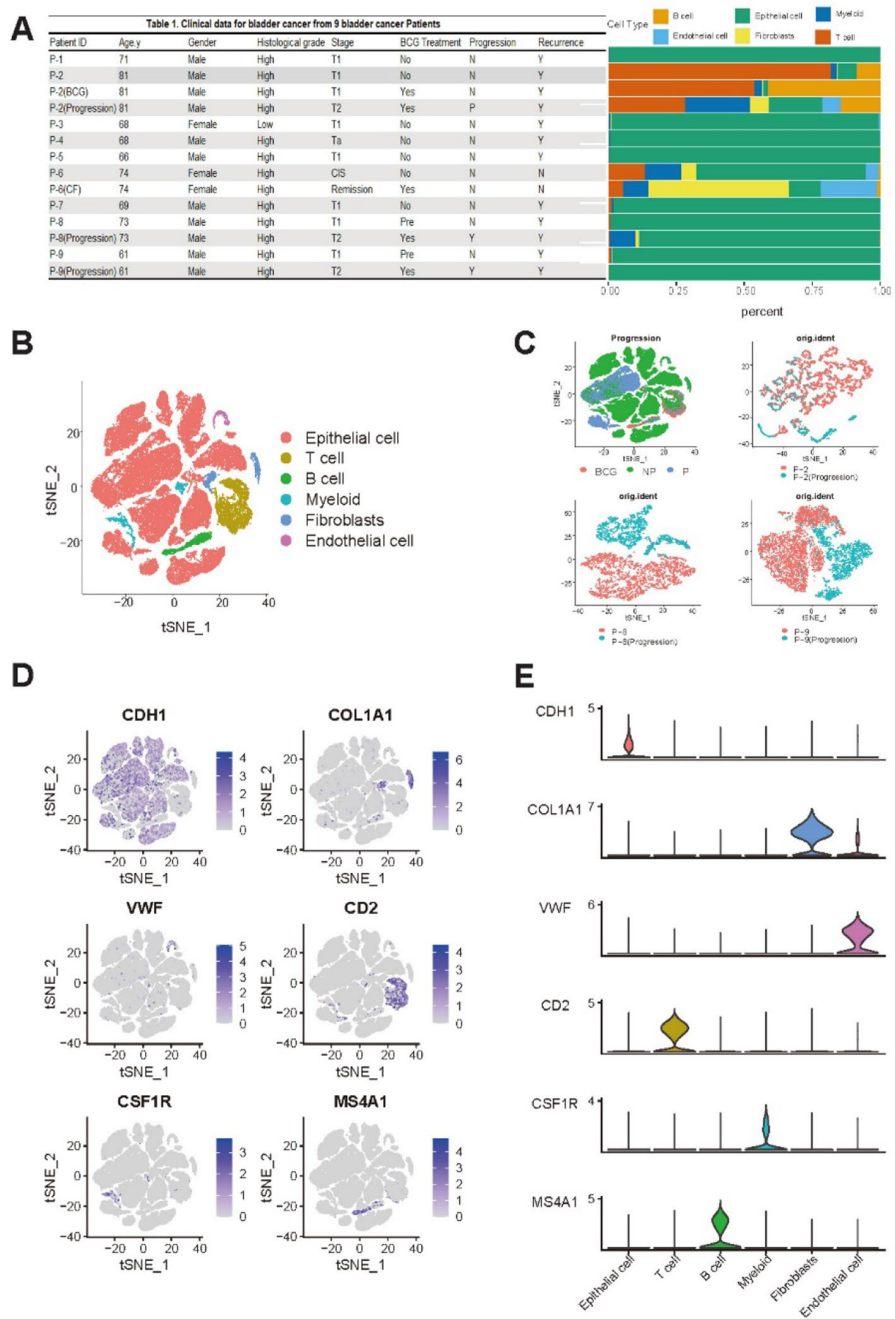
Single-cell transcriptome analysis was performed on samples from 9 bladder cancer patients. **A** Baseline information of clinical features, along with cell abundance in cancer tissues from the 9 patients. **B** tSNE plot of 54,393 cells from the 9 patients, colored by their 6 major cell types. **C** tSNE plot showing cell distribution across progression states and pairwise comparisons of Primary vs. Progression samples for P-2, P-8, and P-9. **D** Expression patterns of marker genes for the six major cell types, displayed using tSNE plots. **E** Violin plots showing expression levels of marker genes across six major cell types

All samples were collected from nine patients with NMIBC at the Seoul St. Mary’s Hospital (College of Medicine, Catholic University of Korea, Seoul, Republic of Korea). Samples included 9 NMIBC bladder primary specimens, 4 BCG-treated specimens, 3 specimens from patients with disease progression after BCG treatment, and 1 cancer cell–free (CF) specimen (Fig. 1A). In particular, the sample labeled “P2” refers to the baseline resection specimen collected prior to BCG therapy, whereas “P2-BCG” denotes the post-treatment specimen obtained after BCG administration. This labeling was used to distinguish baseline and post-treatment samples from the same patient. All procedures were conducted with informed consent from patients and approved by the Institutional Review Board of Seoul St. Mary’s Hospital.

### Single-cell suspension preparation

Frozen tissue was homogenized and nuclei were counted using a LUNA-FL™ Automated Fluorescence Cell Counter. Nuclei were then isolated using flow cytometry. We used the 10X Genomics Chromium Instrument and cDNA synthesis kit (Chromium Next GEM Single Cell 3' Kit v3.1 & Chromium Next GEM Chip G Single Cell Kit) to generate a barcoded cDNA library for single nuclei RNA-sequencing of sorted nuclei. The cDNA library quality was determined using an Agilent Bioanalyzer.

### Single cell RNA preprocessing

Unique molecular identifiers (UMIs) were quantified using Cellranger 6.1.2 (https://support.10xgenomics.com) with a reference transcriptome GRCh38. Subsequent analysis was performed using R (version 4.3.2) and Seurat (version 5.0.1) [12]. Single cells were filtered based on gene counts, retaining cells with 300 to 8000 gene counts per cell (nFeature_RNA) and less than 30% mitochondrial content as part of the initial quality control process. This ensured that only high-quality cells were used in downstream analyses.

Potential doublets were identified and filtered out using the DoubletFinder software (version 2.0.4) [13] with standard parameters. Initially, there were 5,804 cells. After removing the cells classified as doublets, 5,439 cells were used for downstream analysis.

UMI-collapsed read count matrices were processed in Seurat following the standard workflow. All data were then integrated using the FindIntegrationAnchors() function, defining anchor sets based on 2,000 corresponding features between cells selected using the SelectIntegrationFeatures() function, with each section initially analyzed separately before being merged using Seurat's SelectIntegrationFeatures, FindIntegrationAnchors, and IntegrateData functions. Relevant clusters were subsetted using the Seurat subset function for improved annotation, followed by normalization, transformation, principal component analysis (PCA), and clustering of each merged dataset and/or subset as previously described to identify cell types using well-known markers from previous research [12].

### Copy number profiling

To identify large-scale chromosomal copy number variations (CNVs) in single-cell RNA-Seq data of bladder cancers, including amplifications and deletions of whole or large chromosome segments, we used the InferCNV (version 1.10.1) (https://github.com/broadinstitute/inferCNV) software package. This approach can determine the intensity of gene expression by comparing each tumor cell's gene expression to that of reference (cancer-free) cells. We used InferCNV to predict CNAs using the following settings: cutoff = 0.1 (this value was found to generally work well with 10X Genomics and 3ʹ-end sequencing and droplet assays) and leiden_resolution = 0.00005. A heatmap was generated illustrating relative expression intensities across each chromosome. In inferCNV, a non-negative matrix factorization (NMF)-based convolution step is applied to denoise gene expression signals and smooth large-scale patterns. This approach helps to enhance the detection of broad chromosomal gains or losses by reducing gene-level noise while preserving overall CNV structure.

### Analysis of function using hallmark and ssGSEA

We applied single-sample Gene Set Enrichment Analysis (ssGSEA) using the “GSVA” [14] R package to quantify specific scores for various gene sets. Specifically, we calculated cellular senescence scores for progressed patients using hallmark gene sets obtained through GSVA analysis. ssGSEA, an extension of the GSEA method, can compute aggregated enrichment scores for gene sets by ranking gene expression relative to the remaining genes in the genome within progression samples. After correlating ssGSEA results with pseudotime, we visualized the strength of pathway enrichment using a heatmap. We used CIBERSORTx to deconvolute major cell types of bladder cancer in bulk-level transcriptome data.

### Pseudotime analysis

Using UMI count matrices and setting the negbinomial.size() parameter, we created a CellDataSet object in its default configuration. We performed pseudotime analysis using the Monocle [15] R package (version 2.30.1) to determine potential lineage differentiation trajectories. First, we selected a set of ordering genes that showed differential expression between clusters. Monocle then used reversed graph embedding, a machine learning technique, to learn a parsimonious principal graph and reduce high-dimensional expression profiles to a low-dimensional space. Single cells were projected onto this space and ordered into a trajectory with branch points. Cells in the same segment of the trajectory were classified. ssGSEA enrichment scores (e.g., HALLMARK-TGF-BETA-SIGNALING) were mapped onto pseudotime trajectories. Associations between pseudotime and enrichment scores were assessed using Spearman’s rank correlation.

### Cell–cell communication network in the TME

Intercellular communication in bladder tumor samples from the 9 patients was analyzed using the CellChat [16] package. This analysis aimed to understand the landscape of intercellular interactions in the tumor microenvironment. CellChat provided insights into major signaling inputs and outputs among different cell clusters, allowing for visualization of cell–cell communication networks and significant ligand-receptor interactions. These findings were further evaluated for their clinical relevance in predicting prognosis.

NicheNet [17] analysis was performed using the nichenetr package (version 2.0.4). This analysis cam leverage public databases such as KEGG, ENCODE, and Hallmark to conduct a comprehensive examination of cell–cell interactions. Using our cell type–labeled whole atlas Seurat object, we specified malignant cells as the receiver cell type with fibroblasts and macrophages being sender cell types. The condition of interest was progression in nichenet_seuratobj_aggregate. NicheNet was applied to infer interaction mechanisms between fibroblasts, macrophages, and malignant cells, defining niches of interest for each subcluster. For pathway analysis within NicheNet, we used the TGF-β signaling pathway from the hallmark pathway.

### Survival analysis

Kaplan–Meier analyses were conducted to assess survival differences between different risk groups. Survival analysis was performed using the R package survival (version 3.5.7). A two-sided log-rank test was used to compare Kaplan–Meier survival curves.

## Results

### Landscape of single cell nuclei of bladder cancers

Nine patients diagnosed with NMIBC were enrolled in this study. All patients were treated with adjuvant BCG after endoscopic resection. Three patients experienced disease progression. They were classified as "progressors". The remaining six patients who did not experience disease progression were classified as “non-progressors.” Detailed clinicopathological information of these patients is provided in Fig. 1A.

To characterize cell types in the tumor microenvironment (TME) of NMIBC, a total of 14 specimens (P-2, P-8 and P-9 specimens per patients including three post-progression specimens, 1 cell-free specimen) were subjected to snRNA-seq using resection specimens before BCG treatment. A total of 73,979 nuclei were obtained after removing cell doublets (median of 5,804 nuclei, 1,000–8,720 nuclei per patient) and profiled for mRNA quantification. Detailed information on snRNA-seq is available in Supplementary Table S1. Clustering of snRNA-seq revealed six major cell types: 61,837 epithelial cells, 898 endothelial cells, 5,973 T cells, 1,907 B cells, 1,712 myeloid cells, and 1,652 fibroblasts.

A t-distributed stochastic neighbor embedding (tSNE) plot was used to illustrate cell distribution as shown in Fig. 1B. Notably, epithelial cells constituted a significant fraction of cells, indicative of high tumor purity across samples. Moreover, heterogeneity between Progression and NP samples was observed as shown in pairwise tSNE plots (P-2, P-8, and P-9), Primary and progression samples formed well-separated clusters, indicating distinct transcriptional differences (Fig. 1C). The expression of marker genes for each major cell type is depicted in Fig. 1D, including *CDH1* [18] for epithelial cells, *VWF* [19] for endothelial cells, *COL1A1* [20] for fibroblasts, *CSF1R* [21] for myeloid cells, *CD2* [22] for T cells, and *MS4A1* [18] for B cells. Additionally, cluster-specific gene expression of selected markers is presented in Fig. 1E.

### The TGF-β signaling pathway is activated in malignant cells with disease progression

**Fig. 2:**
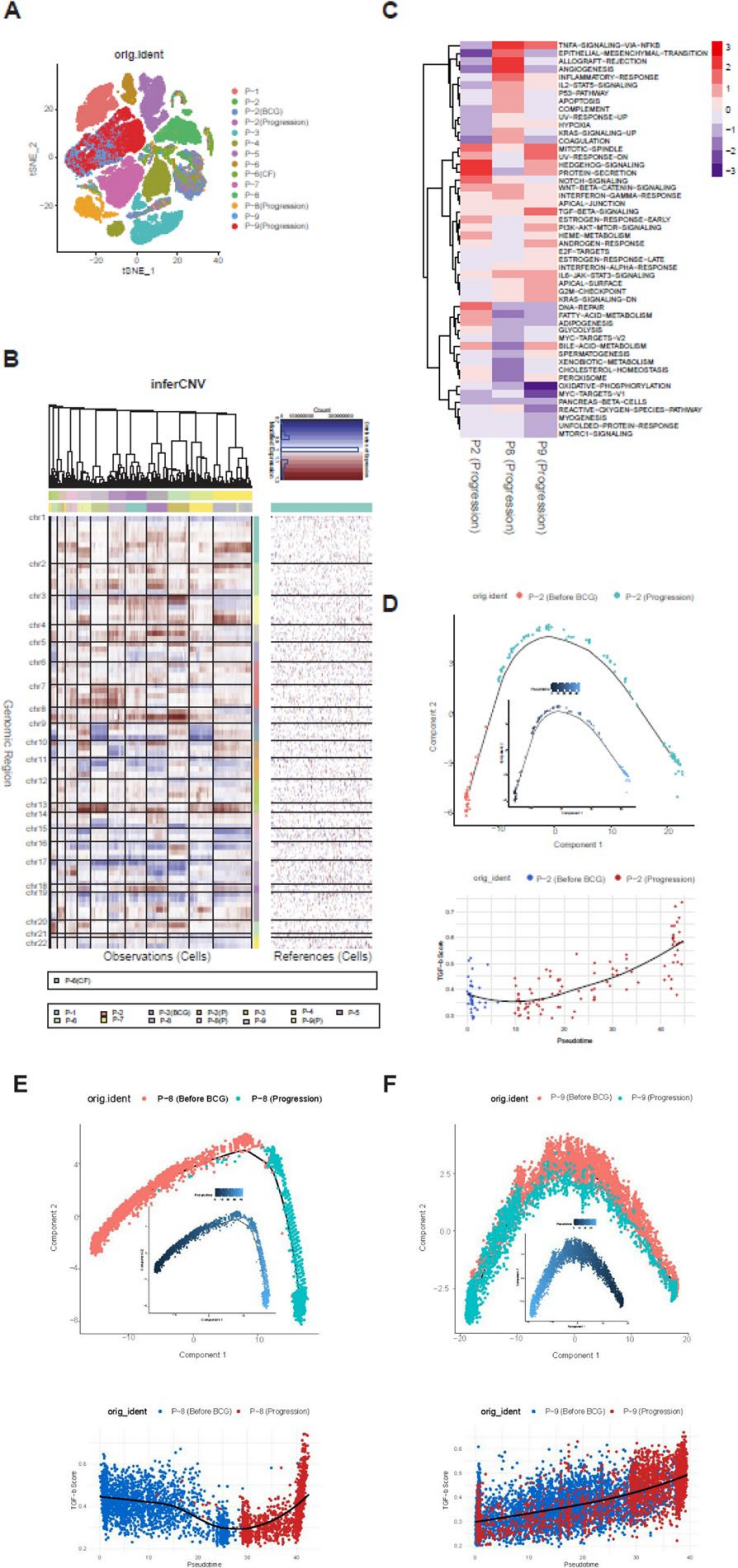
Identification of malignant and defined malignant functions using ssGSEA (**A**) tSNE plot of 61,837 epithelial cells. (**B**) Chromosomal gains and losses in malignant epithelial cells were predicted using inferCNV. (**C**) A heatmap displaying enrichment scores from the ssGSEA analysis and correlation values between the ssGSEA score and monocle pseudotime results of the progression samples by Cell ID. (**D**)-(**F**) Monocle analysis of the development of malignant cells between P-2/P-8/P-9 Progression (Before BCG) and P-2/P-8/P-9 (Progression) with pseudotime, as well as the pseudotime trajectory of TGF-β signaling for P-2/P-8/P-9 Progression (Before BCG) and P-2/P-8/P-9 (Progression)

Epithelial cells were further subject to fine-clustering. tSNE plots of epithelial cells revealed that the clustering of epithelial cells was largely determined with respect to individual patients, indicative of a substantial level of inter-patient heterogeneity of malignant cells (Fig. 2A). We then performed inferCNV to determine copy number alterations (CNA) based on gene expression. Using inferCNV analysis, we identified significant interpatient heterogeneity in inferred CNA profiles across nine bladder cancer malignant cells (Fig. 2B). Notable arm-level gains were observed in chromosomes 1q, 5p, and 20p, while frequent losses were detected in 9p, 11p, 17p, and 19p. Hurst et al. [23] have reported that gains of 1q and 5q and losses of 9p, 11p, 17p, and 19p are associated with disease progression of bladder cancers. In addition, Bellmunt et al. [24] have found that gains in 20p are linked to disease recurrence, while López et al. [25] have identified a connection between gains in 5q and bladder cancer recurrence.

We analyzed malignant cells using longitudinal specimens obtained before treatment and after disease progression. Functional gene sets were identified using ssGSEA with representing sample-specific disease progression using Hallmark pathway. This analysis identified molecular functions enriched in the difference of NES values between Progression and Primary specimens. It highlighted tumor progression-related functions, such as WNT–β-catenin signaling and TGF-β signaling, which are consistently enriched across malignant cells during disease progression (Fig. 2C). This result suggests that TGF-β might play a key progression-related molecular function in disease progression after BCG (Figs. 2D–2F). Pseudo-temporal analysis representing the transition from treatment-naïve to progression samples indicated that each paired sample of malignant cells passed through smooth one-dimensional curves. Specifically, cells from treatment-naïve samples were ordered at early pseudotime points, followed by cells from progression samples at later pseudotime points. This indicates a clear separation in differentiation trajectories of treatment-naïve and progression cells. Results of the ssGSEA analysis of TGF-β signaling along the pseudotime trajectory showed that TGF-β signaling enrichment scores increased progressively from treatment-naïve to progression samples, consistent with the trajectory analysis.

### TME cell subtypes associated with disease progression

**Fig. 3:**
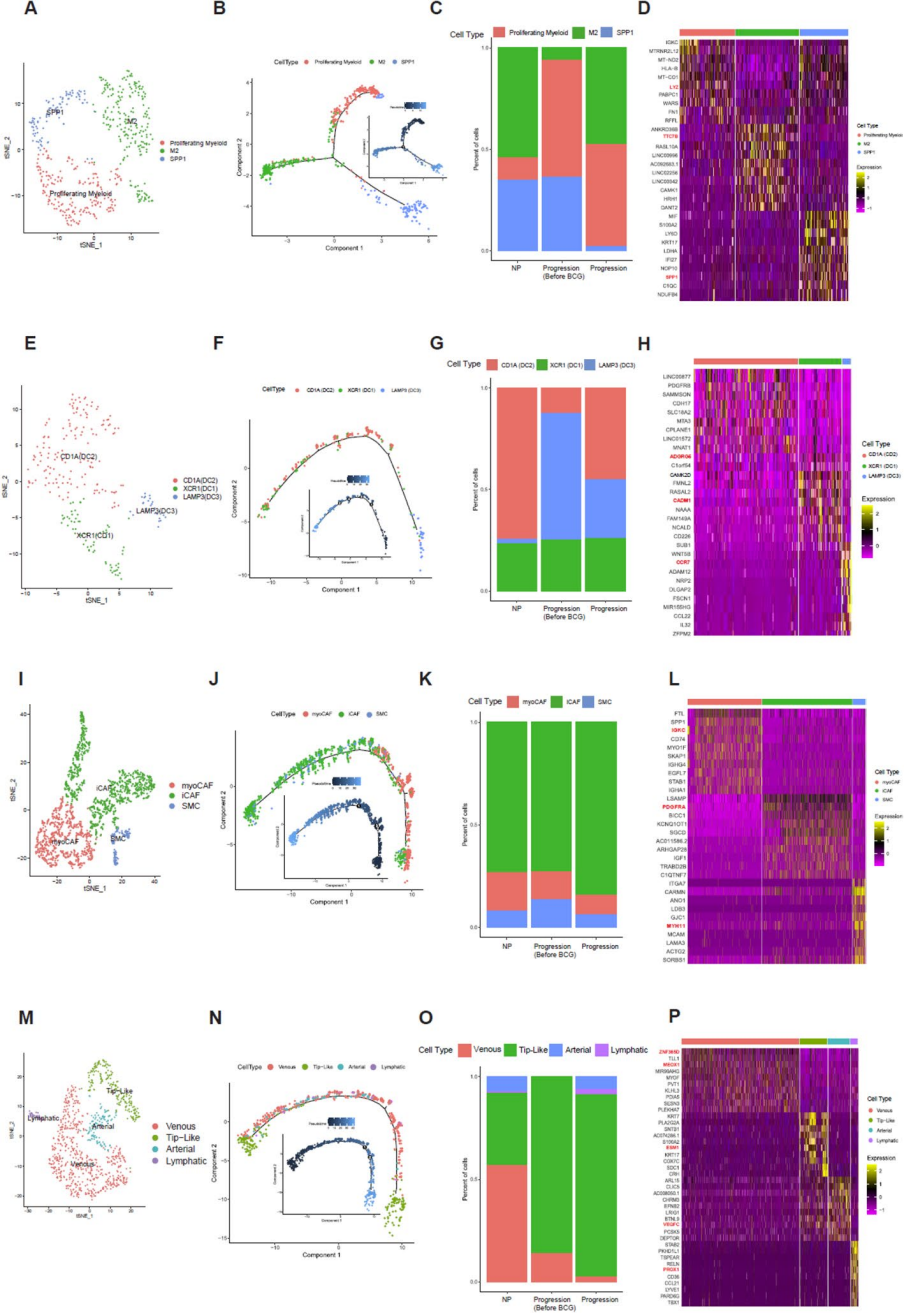
Characterization and profiling of four subtypes. **A** tSNE plot of 508 macrophage cells, showing the composition of three main subtypes. **B** Monocle analysis of the development between subtypes with pseudotime. **C** Cell proportion of subtypes by NP, Progression Before BCG, and Progression. **D** Heatmap of Top 10 marker genes of each subtype. **E** tSNE plot of 245 dendritic cells, showing the composition of 3 main subtypes. **F** Monocle analysis of the development between subtypes with pseudotime. **G** Cell proportion of subtypes by NP, Progression Before BCG, and Progression. **H** Heatmap of Top 10 marker genes of each subtype. **I** tSNE plot of 1,652 fibroblasts, showing the composition of the 3 main subtypes. (**J**) Monocle analysis of the development between subtypes with pseudotime. **K** Cell proportion of subtypes by NP, Progression Before BCG, and Progression. **L** Heatmap of Top 10 marker genes of each subtype. **M** tSNE plot of 898 endothelial cells, showing compositions of 4 main subtypes. **N** Monocle analysis of development between subtypes with pseudotime. **O** Cell proportion of subtypes by NP, Progression Before BCG, and Progression. **P** Heatmap of Top 10 marker genes of each subtype

Sub-clustering of macrophages (Fig. 3A; proliferating, M2 and SPP1 + macrophages) revealed three distinct subtypes. Pseudotime trajectory analysis showed bifurcation into two major branches, with proliferating and some subtypes located in earlier stages, M2 macrophages diverging into another branch, and SPP1 + macrophages appearing in the later phase (Fig. 3B). M2 macrophages were relatively more abundant in the Progression group than in the Progression-Before BCG group. Proliferating myeloid cells showed a gradual increase from NP to Progression-Before BCG and remained elevated in the Progression group, whereas SPP1 + macrophages exhibited a consistent decrease across groups, with the lowest proportion observed in the Progression group (Fig. 3C). The top 10 marker genes of macrophage subtypes are shown in Fig. 3D. The LYZ gene was identified as a marker gene for proliferating myeloid cells [26, 27]. In previous studies on bladder cancer, LYZ has been used as a myeloid cell marker. Moreover, Gu et al. have demonstrated a significant association between these cells and proliferation. Regarding the TTC7B gene, He et al. have found notable correlations between its expression and infiltration levels of various immune cells, including M2 macrophages [28].

Sub-clustering of dendritic cells (Fig. 3E; DC1, DC2, and DC3) demonstrated sequential differentiation. The pseudotime trajectory (Fig. 3F) followed an order from DC3 (LAMP3 +) at the earliest stage, through DC2 (CD1A +), and finally to DC1 (XCR1 +) at the latest stage, highlighting dynamic changes in cell states. For dendritic subtypes, CD1A (DC2) cells showed only a modest and variable increase in the Progression group compared with NP and Progression-Before BCG, without a consistent trend across patients (Fig. 3G). Dendritic cells are known for their roles in antigen presentation and immune response orchestration. They exhibited CADM1 expression, particularly in XCR1 (DC1), as highlighted by our DEG results where CADM1 emerged as a marker (Fig. 3H). To understand CADM1's function [29], future research needs to be conducted on tumor progression where CADM1's role has been extensively studied. Kang et al. have found that the ADGRG5 gene in CD1A (DC2) is associated with dendritic cells, showing a stronger correlation with ADGRG5 expression than in other types of immune cells tested [30]. In our study, ADGRG5 was identified as a marker in DC2. In DCs, CCR7-dependent migration from peripheral tissues to lymphoid tissues plays a critical role in host defense against pathogens and immune tolerance maintenance [31]. This migratory process is governed by complex intracellular signaling pathways that can regulate both DC movement and inflammatory responses. Our analysis identified CCR7 as a marker gene specifically in LAMP3-expressing DCs (DC3), as shown in our differential gene expression data (Fig. 3H).

Fibroblast subtypes (Fig. 3I; myoCAF, iCAF, and SMC) also showed distinct developmental patterns. Pseudotime analysis (Fig. 3J) indicated that myoCAF and iCAF dominated the early phase, while SMC and iCAF became predominant later, with the trajectory culminating in iCAF and SMC states. Results revealed a higher proportion of myoCAF cells in the Progression Before BCG group compared with the Progression group. Additionally, SMC cells showed a consistent proportion across all groups. Notably, iCAF cells maintained the highest proportion in all groups, although they were slightly reduced in the Progression group compared with NP and Progression Before BCG groups (Fig. 3K). Both iCAF and myoCAF showed similar cell proportions, although iCAF demonstrated a stronger correlation with reduced survival rates. The top 10 marker genes of fibroblast subtypes are displayed in Fig. 3L.

Sub-clustering of endothelial cells (Fig. 3M; venous, tip-like, arterial, and lymphatic) revealed further heterogeneity. Pseudotime trajectory analysis (Fig. 3N) indicated that venous and tip-like cells appeared in early stages, arterial and lymphatic cells arose but later disappeared, leaving tip-like cells predominant at the end. The distribution of endothelial cell subtypes within each group is illustrated in Fig. 3O. In the NP group, venous cells constituted the highest proportion, followed by tip-like cells. Arterial and lymphatic cells were present in relatively smaller proportions. In the Progression Before BCG group, tip-like cells dominated almost entirely, with a small presence of venous cells. Arterial and lymphatic cells were nearly absent. In the Progression group, tip-like cells also constituted the highest proportion, although the proportion of venous cells was significantly reduced compared with levels in NP and Progression-Before BCG groups. Additionally, proportions of arterial and lymphatic cells slightly increased. The top 10 marker genes of endothelial subtypes are shown in Fig. 3P. In humans, loss-of-function mutations in VEGFC or VEGFR3 can lead to lymphedema, while the application of recombinant VEGFC can stimulate robust lymphangiogenesis in adults, suggesting its therapeutic potential for lymphedema and tissue repair.

To distinguish the function of each subtype, we performed hallmark pathway analysis using ssGSEA and selected the top 10 hallmark pathways. As shown in Supplementary Fig. 1A, Proliferating Macrophage subtype was enriched in the EMT and MYC-TARGETS-V2 pathways, while the M2 subtype was enriched in the PI3K-AKT-mTOR-SIGNALING and TGF-BETA-SIGNALING pathways. Additionally, the *SPP1* subtype was enriched in the MYC-TARGETS-V1, HYPOXIA, and DNA-REPAIR pathways. The population of dendritic cells was clustered into three subtypes (DC1, DC2, and DC3), although they were not clearly separated in ssGSEA Hallmark pathway. However, DC2 tended to be enriched in the DNA-REPAIR pathway, while DC showed a tendency to be enriched in the EMT pathway (Supplementary Fig. 1B). Additionally, fibroblasts were divided into three sub-clusters (myoCAF, iCAF, and SMC). The myoCAF subtype was enriched in immune-related pathways and the INFLAMMATORY-RESPONSE pathway, whereas iCAF and SMC subtypes were not clearly distinguished as shown in Supplemantary Fig. 1C. The venous subtype, a subset of endothelial cells, was enriched in immune-related pathways and the INFLAMMATORY-RESPONSE pathway, while the Tip-like sub-cluster was enriched in the MYC-TARGETS-V1 pathway. Additionally, the arterial subtype was enriched in the COMPLEMENT and ANGIOGENESIS pathways, whereas the Lymphatic sub-cluster was enriched in the NOTCH-SIGNALING pathway, as shown in Supplementary Fig. 1D.

### Cell–cell interactions across malignant cells and TME cells

**Fig. 4:**
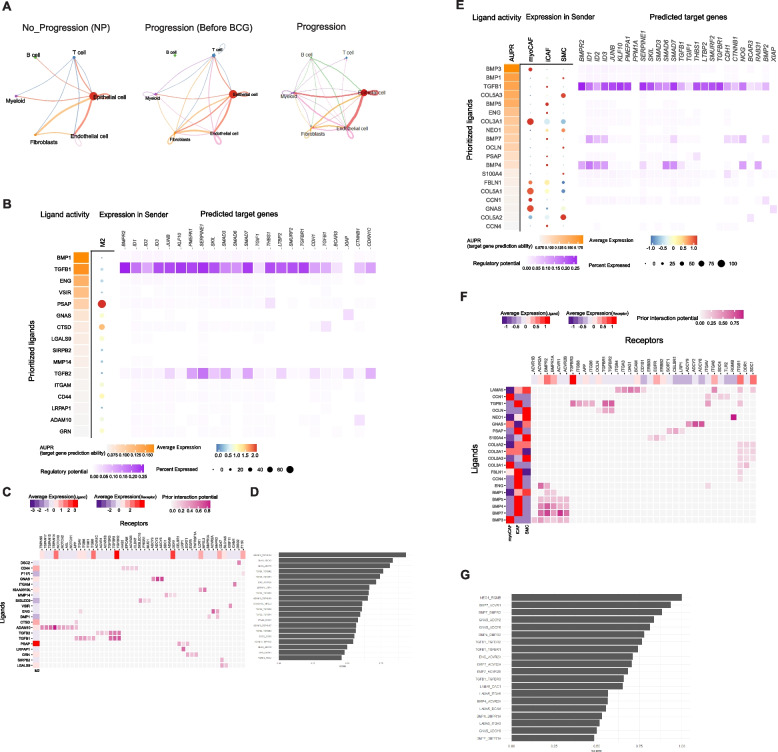
Estimation of cell–cell communication and interactions between malignant and macrophage/fibroblasts using TGF- β signaling pathway geneset. **A** Interaction plot for major cell types. The strength of communication was assessed via CellChat across NP, Progression Before BCG, and Progression. **B** Using the TGF- β signaling pathway geneset in Nichenet, interactions between malignant cells and Macrophages were examined. **C** A heatmap displays active ligand-receptor pairs, highlighting interaction potential between receptors on malignant receiver cells and ligands on Macrophage sender cells. **D** Top 30 ligand-receptor (L-R) pairs ordered by interaction potential score. **E** Interactions between malignant cells and fibroblast using the TGF- β signaling pathway geneset in Nichenet. **F** A heatmap displays active ligand-receptor pairs, highlighting interaction potential between receptors on malignant receiver cells and ligands on fibroblast sender cells. **G** Top 30 ligand-receptor (**L**-**R**) pairs ordered by interaction potential score

To investigate differences in cell–cell communication among various cell subpopulations, we analyzed interaction numbers and interaction strength using CellChat [16]. Our study included three conditions: No Progression (NP), Primary Before BCG, and Progression. Results in Fig. 4A illustrate variations in interaction patterns across these conditions. In the NP condition, interactions were relatively sparse, with notable communication occurring primarily between epithelial cells and other cell types. In the Primary condition (Before BCG), the interaction network became more complex, indicating increased communication among T cells, B cells, myeloid cells, and other subpopulations. The Progression condition showed the most extensive and robust interactions, suggesting a heightened level of cell–cell communication. We conducted a pathway-based ligand-receptor interaction analysis between TME cells. Results (Fig. 4A) demonstrated dynamic changes in cell–cell communication networks across different conditions, emphasizing the importance of epithelial cell interactions in NP, Progression Before BCG, and Progression. Notably, the interaction strength between epithelial cells and myeloid cells, as well as fibroblasts, increased from Progression Before BCG to Progression.

According to Browaeys et al. [17], a validation study can substantiate the proposed methodology, illustrating that the final prior model of ligand–target interactions can be broadly applied to various biological systems, thereby supporting the applicability of Nichenet to a wide range of biological contexts. Consequently, we employed the TGF-β signaling pathway identified in Fig. 2 to analyze Nichenet. We defined the gene set of interest as those genes within the receiver cell type that are likely to be influenced by the cell–cell communication event.

Results shown in Fig. 4A revealed that the interaction strength between epithelial cells and myeloid cells, as well as fibroblasts, increased from the Progression Before BCG stage to the Progression stage. Consequently, we analyzed ligand-receptor interactions during muscle regeneration in Progression samples using NicheNet. By applying stringent cutoffs, we identified differentially expressed ligands predicted to interact with receptors on malignant cell (top 20 by differential expression). We compiled a list of ligand-receptor pairs expressed in relevant cell types and predicted them to act upstream of TGF-b signaling specific genes. To narrow down the extensive list of candidates, we focused on ligands with high activation scores for TGF-b signaling genes and complementary targets in macrophages (Figs. 4B–4C) and fibroblasts (Figs. 4E–4F). In addition, we identified the top 30 ligand-receptor pairs in macrophages (Fig. 4D) and fibroblasts (Fig. 4G).

### Establishment and validation of prognostic significance marker genes

**Fig. 5:**
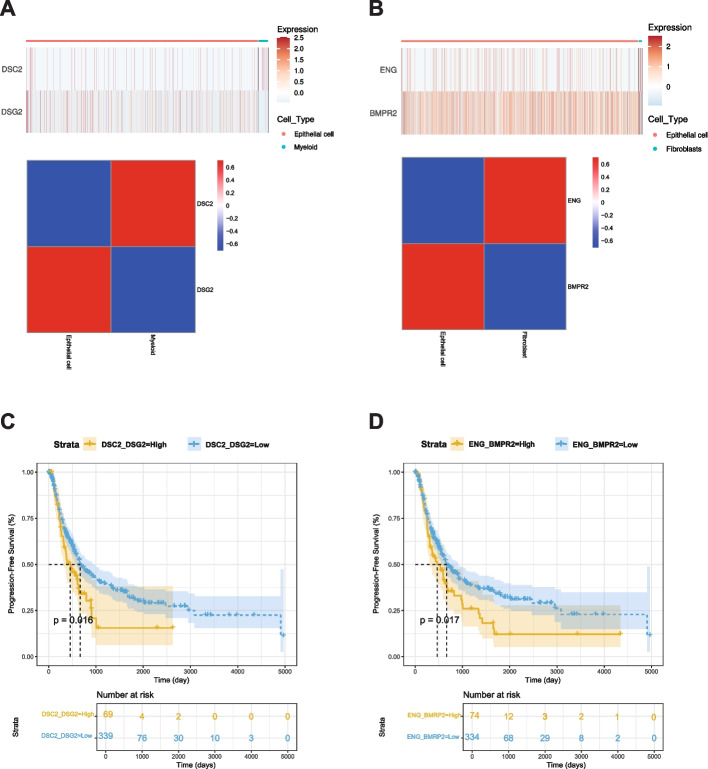
Kaplan–Meier analysis of BLCA progression-free survival (PFS) associated with ligand–receptor pairs. **A** Expression heatmap and correlation matrix of DSC2 (myeloid cells) and DSG2 (epithelial cells). **B** Expression heatmap and correlation matrix of ENG (fibroblasts) and BMPR2 (epithelial cells). **C** Kaplan–Meier PFS curve comparing high vs. low expression of the DSC2–DSG2 pair (*p* = 0.016). **D** Kaplan–Meier PFS curve comparing high vs. low expression of the ENG–BMPR2 pair (*p* = 0.017)

Using TCGA data, we employed CIBERSORTx to estimate proportions of M2 macrophages and fibroblasts. As shown in Figs. 4D and 4G, top 30 ligand-receptor pairs were identified the. Based on these results, we examined the expression of the DSC2(L)-DSG2(R) pair in epithelial and myeloid cells and the expression of the ENG(L)-BMPR2(R) pair in epithelial and fibroblast cells from our snRNA-Seq data. We then analyzed their survival associations in TCGA BLCA data. Results are shown in Fig. 5. Patients with a higher average proportion of M2 macrophages and elevated expression of the DSC2(L)-DSG2(R) pair demonstrated a significant difference in survival with a *p*-value of 0.016 (Fig. 5A). Similarly, patients with a higher average proportion of fibroblasts and increased expression of the ENG(L)-BMPR2(R) pair also showed a significant survival difference, with a *p*-value of 0.017 (Fig. 5B). According to Nakauma-González et al. [32], DSC2 and DSG2 in bladder cancer are markers of intratumoral genomic and immunologic heterogeneity, particularly evident through squamous differentiation. This morphological heterogeneity can serve as a biomarker for intrinsic immunotherapy resistance in bladder cancer patients.

## Discussion

This study investigated the TME of NMIBC in nine patients treated with BCG after endoscopic resection using snRNA-seq. Patients were categorized into progressors and non-progressors based on their disease progression. Longitudinal pairs from three progressors (BCG-naïve and progression specimens) were compared to reveal transcriptional changes associated with disease progression after BCG treatment. Among the six major cell types in NMIBC TME, malignant cells were our focus. Transcriptional upregulation was noted for TGF-β signaling pathway-related genes during disease progression. This observation was supported by pseudotime analyses where the inferred pseudotime showed correlated with the expression level of genes belonging to TGF-β signaling pathway. The TGF-β pathway is known to play a pivotal role in cancer progression, where TGF-β signaling often becomes dysregulated during disease progression, contributing to tumor growth and metastasis [33]. In our study, increased TGF-β signaling has been observed during disease progression where cell-level enrichment scores of the pathway are elevated along the pseudotime represented by trajectory of disease progression. In addition, cellchat analysis results of three groups (NP, Before BCG, Progression) revealed cell–cell interactions of major cell types. Compared with Progression Before BCG and Progression, epithelial cells, myeloid and fibroblasts indicated the strength and frequency of their interactions.

Moreover, enhanced TGF-β signaling has been reported to influence cell–cell communication between malignant cells and other TME components, including myeloid cells and fibroblasts. In our dataset, we observed that TGF-β signaling appeared more pronounced in a subset of progression samples. This may suggest that the pathway could not only contribute to the malignant potential of cancer cells but also facilitate communication with the surrounding NMIBC TME. For instance, the DSC2(L)–DSG2(R) interaction between epithelial and myeloid cells was detected in our analysis, and evaluation of independent datasets (TCGA) indicated that such interactions may also be assessed at the bulk expression level with possible relevance to patient survival. Similarly, the ENG(L)–BMPR2(R) gene pair may represent one of the interactions between epithelial and fibroblast populations, potentially linking to clinical outcomes (PFS). While these observations highlight possible roles for TGF-β–associated interactions during progression after BCG treatment, we acknowledge that enrichment was observed in only a subset of patients. Thus, further validation across larger cohorts will be required to confirm the extent to which these interactions broadly contribute to NMIBC progression.

Disease progression-associated cellular dynamics confirmed previous studies, the proportion of *SPP1* Myeloid and Proliferating cells is increased in tumors compared to that in normal tissues [34]. For dendritic cells, more *LAMP3*(DC3) cells were observed in the Progression Before BCG group than in the Progression group, while *XCR1*(DC1) cells stayed about the same across all groups. Furthermore, the proportion of cDC2 cells varies across cancer types. During their differentiation into *LAMP3* + cDCs, their immunosuppressive functions are enhanced, providing valuable insights for the development of cancer immunotherapy strategies [35]. iCAF and mCAF exhibited similar characteristics in bladder cancer tissues. However, iCAF is strongly associated with decreased survival rates in bladder cancer patients, suggesting that iCAF plays a more critical role. Tip-like ECs were predominantly found in tumor tissues across various cancer types. They were rarely observed in normal tissues. This suggests that Tip-like ECs might be associated with activation of angiogenesis in tumor tissues. Additionally, the cell proportion of Tip-like ECs is higher in tumors [33].

Jia et al. have reported that one type of myCAF expresses immunoglobulin genes (*IGKC*) [36]. Our study clearly demonstrated that the *IGKC* gene could serve as a specific marker for myoCAFs. It was also used in our study to annotate myoCAFs. Chen et al. have observed that *PDGFRA* + fibroblasts exhibit robust expression of cytokines and chemokines [26]. They also reported that *PDGFRA*, a specific marker for iCAFs, correlated significantly with poor overall survival in bladder cancer patients. Similarly, our differential gene expression analysis of bladder cancer fibroblast subtypes identified *PDGFRA* as a marker specifically in iCAFs. Our study revealed that *MYH11* emerged as a marker gene for SMCs through DEG analysis. This finding aligned with a previous research study, which reported that SMCs could transition into fibromyocytes/FCs via an *Lgals3* + transitional state. That study used Monocle 3 to define *MYH11*-expressing contractile SMCs as the starting point of phenotypic modulation, predicting that transitional SMCs would adopt either a fibromyocyte or FC fate [37]. Similarly, our results indicated that myoCAFs originated in the early state, with SMCs passing through an intermediate state before transitioning to FCs, while iCAFs were present in the late state. This suggests that SMCs from different vascular beds can adopt a common transitional state before reaching ECM-rich phenotypes. According to this result, iCAF emerged as pseudotime progressed from an early stage to a late stage. Figure 3K shows that the proportion of iCAF is higher in Progression.

According to Koshiba et al. [38], genes such as *AGBL4* and *ZNF385D*, although not directly involved in related metabolic pathways, are linked to plasma phospholipids and lipid-related diseases. Variants near *ZNF385D* are linked to increased blood levels and a higher risk of arterial and venous thrombosis. Additionally, SNPs in *ZNF385D* are connected to language-based learning disabilities and impairments. These findings suggest that *ZNF385D* might play an unknown role in lipid metabolism. Similarly, in our study, *ZNF385D* emerged as a marker in the venous subtype. Douville et al. have shown that the homeodomain transcription factor MEOX1 can regulate vascular cell proliferation by activating the expression of cyclin-dependent kinase inhibitors, specifically p21CIP1/WAF1 and p16INK4a [39]. Notably, *MEOX1* activates p16INK4a through DNA binding, while it induces p21CIP1/WAF1 expression through a mechanism independent of DNA binding. Our study identified the *MEOX1* gene as a marker in the venous subtype through DEG analysis.

Rocha et al. [40] have reported that *ESM1* is intricately involved in both modulation and targeting of VEGF signaling within endothelial cells, exerting significant influence over processes such as angiogenesis, inflammation, and vascular permeability. These attributes underscore its potential therapeutic relevance. Notably, heightened expression of *Esm1* is present in retinal endothelial tip cells and in tumor endothelium, establishing it as a valuable marker for neoangiogenesis. In our study, the *ESM1* gene was identified as a marker in the tip-like subtype through DEG analysis. *VEGFC* is a key regulator within the VEGF family, influencing both blood vessel growth (angiogenesis) and lymphatic vessel growth (lymphangiogenesis) [41]. In humans, loss-of-function mutations in either *VEGFC* or *VEGFR3* can lead to lymphedema. Additionally, recombinant *VEGFC* application can stimulate robust lymphangiogenesis in adults, suggesting its therapeutic potential in managing conditions such as lymphedema and tissue repair. *PROX1* plays a critical role in lymphatic endothelial cell identity by regulating a newly identified enhancer element [42]. This evolutionarily conserved enhancer can bind key transcriptional regulators. Furthermore, mutant mice exhibited altered gene expression profiles, showing reduced markers of lymphatic endothelial cells and increased markers associated with hemogenic endothelium. These findings suggest that *PROX1* not only controls lymphatic endothelial cell identity, but also suppresses the hemogenic potential of these cells. Our results indicate that *ESM1* can regulate VEGF signaling in endothelial cells, influencing angiogenesis and vascular permeability. *ESM1* was identified as a marker for tip-like endothelial cells. *VEGFC* and *PROX1* play key roles in lymphangiogenesis and lymphatic endothelial identity, with therapeutic potential for lymphedema and tissue repair, respectively.

These findings may provide preliminary insights with potential clinical implications. Upregulation of TGF-β signaling and associated cell–cell interactions within the TME were observed in a subset of progression cases, suggesting that these features could serve as hypothesis-generating biomarkers of BCG treatment response. Patients exhibiting heightened TGF-β signaling might be at increased risk of progression despite BCG therapy, highlighting the need for further validation in larger, independent cohorts. In addition, ligand–receptor gene pairs such as DSC2–DSG2 and ENG–BMPR2 were identified as possible mediators of epithelial–immune and epithelial–fibroblast interactions. These observations should be regarded as exploratory and require extensive functional and clinical validation before any therapeutic applications can be considered.

These insights into the impact of the TGF-β signaling pathway and specific cell–cell interactions on BCG treatment response could help clinicians optimize treatment strategies for individual patients, thereby potentially improving treatment outcomes.At the same time, it is important to note that pseudotime analysis of malignant cells should be interpreted with caution, as clonal evolution and genetic heterogeneity may limit the extent to which pseudotime directly reflects chronological progression. Thus, our findings provide relative transcriptional dynamics rather than absolute temporal order.

This study has several limitations. First, the sample size was relatively small (*n* = 9 total patients, including only three paired progression cases), which may limit the generalizability of our findings. Second, external validation and functional assays were not performed, and thus the observed associations should be interpreted with caution. Third, our analyses of cell–cell communication relied on computational inference tools such as CellChat and NicheNet, and not yet experimentally confirmed. Limitations inherent to snRNA-seq technology, including the lack of cytoplasmic transcript capture, may have influenced the transcriptional profiles. Finally, although TCGA BLCA data were incorporated as supportive evidence, this cohort is composed primarily of MIBC rather than NMIBC cases and lacks sufficient clinical covariates for adjusted analyses; accordingly, these findings should be regarded as exploratory.

## Supplementary Information

Supplementary Material: Figure S1.

Supplementary Material: Table S1.

## Data Availability

The snRNA-seq data generated in this study, including the code and annotated data, are available on GEO under accession number GSE270296.
