# Supplementary figures and images for: TGF-β signaling and tumor microenvironment dynamics in bladder cancer progression post-BCG therapy: a longitudinal single-nucleus RNA-seq study
Source: BMC Cancer. 2025 Nov 10;25:1735. doi: 10.1186/s12885-025-15079-8 (PMC12599029; doi:10.1186/s12885-025-15079-8)

A

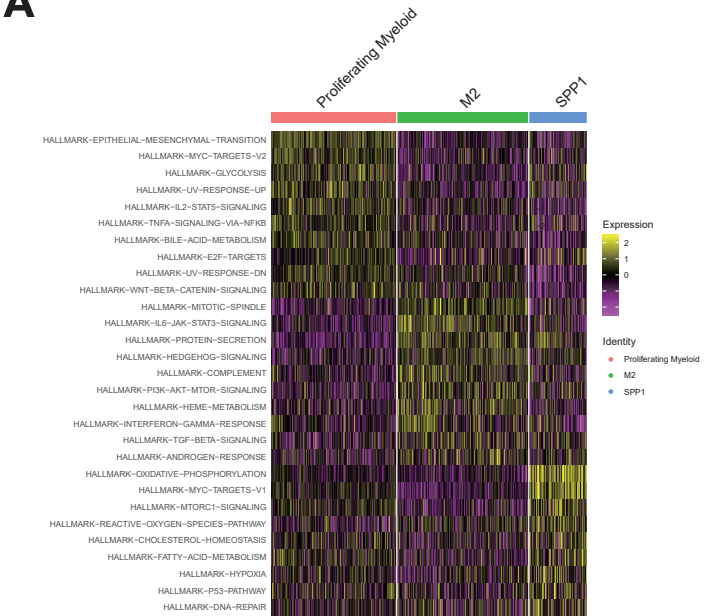

B

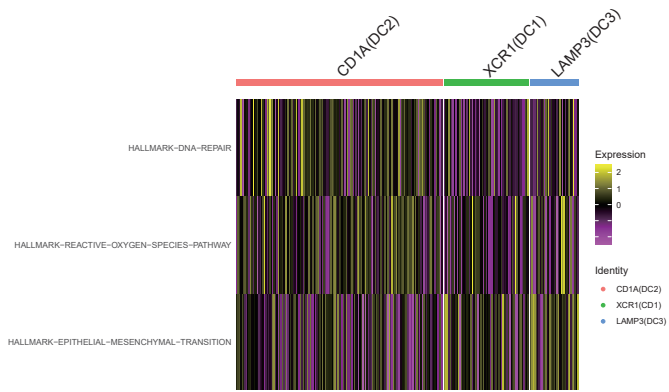

C

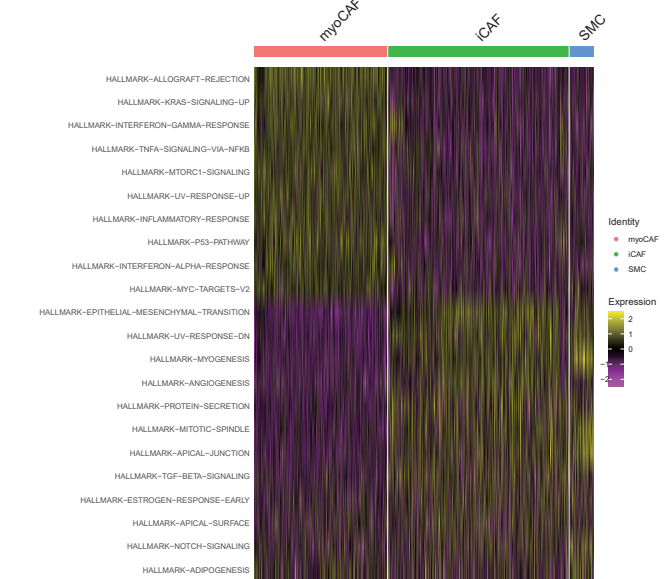

D

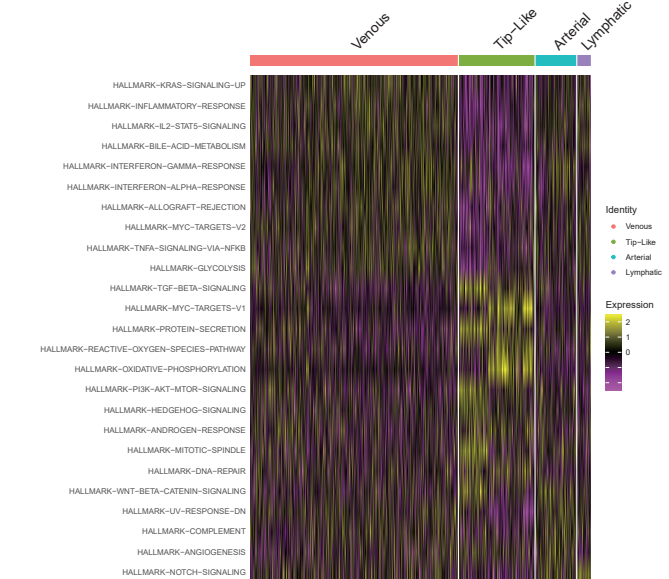

Supplement: Supplementary file 1 — Supplementary Material: Figure S1. [file 12885_2025_15079_MOESM1_ESM.pdf]
